# Lipid-lowering medication reduces more target lesion than non-target lesion revascularizations: a cohort study

**DOI:** 10.1080/07853890.2026.2625586

**Published:** 2026-02-12

**Authors:** Elina Mantyniemi, Mitja Laaperi, Pasi Karjalainen, Juha Sinisalo

**Affiliations:** ^a^ Heart and Lung Center, Helsinki University Hospital and University of Helsinki, Finland; ^b^ Lääperi Statistical Consulting, Helsinki, Finland

**Keywords:** acute coronary syndrome, myocardial infarction, STEMI, NSTEMI, UAP, target lesion, non-target lesion, PCI, statin

## Abstract

**Background:**

Factors related to the very long-term progression of atherosclerosis after stent implantation remain unclear.

**Aims:**

To define factors predicting disease progression as either target lesion (TL) or a new site target lesion (NTL) coronary stenosis.

**Methods:**

In a tertiary hospital, between June 2006 and March 2008, a prospective cohort registry of consecutive patients assigned to a coronary angiogram (*n* = 5294) was collected. Patients who underwent percutaneous coronary stenting (*n* = 1970 patients) were followed for ten years. Of them (1335/67.8%) had acute coronary syndrome (ACS) and (635/32.2%) chronic coronary syndrome (CCS). New ischemic lesions were categorized as TL or NTL.

**Results:**

During a median of 9.5 years follow-up, 639 (32.4%) patients had a cardiovascular event, including 253 (12.8%) cardiovascular deaths. Repeat percutaneous coronary interventions were more frequent in CCS than in ACS patients (144 (22.7%) vs. 204 (15.3%), *p* < 0.001). Of all patients, 137 (39.4%) had TL and 211 (60.6%) had NTL (*p* = 0.209 between TL- vs. NTL-groups). However, TL occurred earlier than NTL (1.2 years, interquartile range [IQR] 0.4–6.2 vs 4.3 IQR, 1.6–7.3 years, *p* < 0.001). Good adherence to statin medication (>80% of time) during the whole follow-up period lowered the odds of TL revascularization compared to NTL revascularization (odds ratio [OR] 0.45, (95% confidence interval [CI] 0.21–0.95, *p* = 0.036)). Diabetes [OR 2.07, (CI 1.04–4.15, *p* = 0.038) and 10 mm increase in stent(s) length (OR 1.26, (CI 1.02–1.55) *p* = 0.031) increased the odds of TL revascularization compared to NTL.

**Conclusions:**

Statin medication provided better protection from TL compared to NTL.

## GRAPHICAL ABSTRACT

**Figure:**
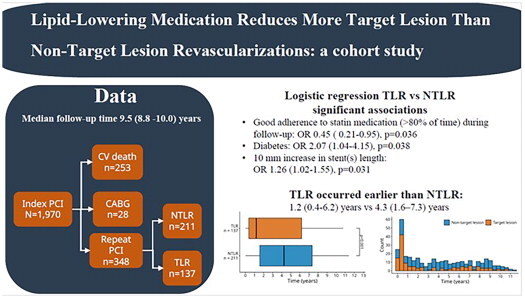

## Introduction

During the first year after coronary stenting ischemic events are mostly related to stent thrombosis or restenosis [1–3]. The rate of stent failure declines after one year to 1–2% per year [3–6]. The rate of stent-related repeat revascularization appears to be higher among CCS patients compared to patients presenting with ST-elevation myocardial infarction (STEMI) [7]. While the development of drug-eluting stents (DES) has reduced the incidence of in-stent restenosis [1,5,6,8–10], patients still experience recurrent ischemic events due to either stent-related complications or the natural progression of atherosclerotic disease. The role of procedural factors such as stent length, vessel size, calcification, and bifurcation lesions in long-term outcomes after percutaneous coronary intervention (PCI) has been widely studied [1,3,5,6,11]. However, less is known about the long-term progression of coronary artery disease.

One mechanism underlying very late stent restenosis has been described as in-stent neoatherosclerosis. Pathophysiological pathways differ to some extent from atherosclerotic process in native vessels. Atherosclerotic plaque formation involves lipoproteins, particularly low-density lipoprotein cholesterol (LDL-C), which is considered the main risk factor for atherosclerotic disease. Transcytosis of LDL-C particles into the tunica intima of the arterial wall triggers an immune response, leading to smooth muscle cell proliferation, extracellular matrix accumulation, and the formation of atherosclerotic plaque. Intensive lipid-lowering treatment has been shown to impede plaque progression and lower the risk of cardiovascular events [12]. Early in-stent restenosis is thought to result from mechanical disruption of the endothelial lining during PCI, which serves as the initiating factor [13]. Findings from intravascular imaging and histological studies suggest that chronic inflammation and endothelial dysfunction contribute to late in-stent neoatherosclerosis, ultimately resulting in very late stent restenosis. In-stent neoatherosclerosis occurs earlier after DES implantation than after BMS implantation, which may reflect differences in the underlying pathophysiological mechanisms [14].

Furthermore, previous studies indicate that while DES substantially reduced in-stent restenosis (ISR) burden compared to bare metal stent (BMS), in-stent neo-atherosclerosis appears to be more common after DES implantation than after BMS implantation [14,15]. This phenomenon has been explained by multiple mechanisms, including drug-driven suppression of neo-intimal growth and incomplete re-endothelization. There are implications that high low-density lipoprotein cholesterol concentration may be independently related to neo-atherosclerosis progression [16,17]. This suggests that good adherence to lipid-lowering medication could prevent very late stent restenosis.

In this article, we aim to define factors related to new ischemia-causing stenosis in the previously stented target lesion (TL) *vs*. new lesions (NTL), with a particular focus on statin adherence, given our extensive follow-up period and access to medication purchase data.

## Methods

### Patients and study design

The prospective Corogene (Genetic Predisposition of Coronary Artery Disease) cohort was collected in Helsinki University Central Hospital, Finland, between March 2006 and March 2008, consisting of 5294 consecutive patients assigned to coronary angiography [18]. A subset of patients who underwent percutaneous coronary stenting (*n* = 1970) was selected for this study. The primary indication for angiography was more often ACS (*n* = 1335) than CCS (*n* = 635). Patients with a history of coronary artery bypass grafting (CABG) were excluded. Patients with previous PCI were included in the study; however, restenosis in those lesions were not included. Patients who did not receive a stent on the index procedure were excluded from the analysis.

Following the index procedure, patients were monitored for up to ten years or until the occurrence of PCI, CABG or death. Ischemia-driven revascularizations, indicated by recurrent angina or myocardial ischemia during follow-up, were systematically analyzed. Subsequent angiograms were compared with the index procedure to define the location of a new ischemic lesion, that is, TL or NTL. Segments 5 mm proximal and 5 mm distal to the stent were considered as stent affected, as proposed by the Academic Research Consortium [19] and were included in stent lesion. Stenosis outside this range were considered new lesions. Stent(s) used in the procedure were bare metal stents or drug-eluting stents based on the decision of the invasive cardiologist. The combined stent length is reported without possible overlapping for multiple stents. Patients who did not undergo a subsequent PCI after the index procedure were excluded, including those who experienced an ischemic event but were managed conservatively or underwent CABG.

National registries in Finland can be merged at the patient level with a unique social security number. This enables the acquisition of data on medication purchases from The Social Insurance

Institution of Finland (SII), follow-up data from the Hospital Discharge Registry of the Finnish

(HILMO) Institute for Health and Welfare based on ICD-codes and causes of death from Statistics of Finland [20]. The validity of the diagnostic codes has been studied previously [21]. The follow-up started in March 2006 and January 2006 for SII’s medication purchase data and HILMO’s discharge data, respectively. The follow-up for these data registries lasted until the end of December 2017 or until CABG or the patient’s death, whichever occurred first.

Baseline characteristics, clinical, laboratory, and angiographic findings were collected from the hospital records. Baseline clinical characteristics included traditional risk factors, including age, sex, previous or current smoking, chronic kidney disease, hypertension, diabetes, obesity and atrial fibrillation. Echocardiograms were reviewed when available. Biomarkers, including N-terminal prohormone B-type natriuretic peptide (NT-proBNP), lipoprotein (a) Lp(a), high-sensitivity C-reactive protein (hCRP) and cystatin-C values, were incorporated into the analysis. Angiographic data was evaluated by two interventional cardiologists (M.E and S.J) independently. Analyses were based on the first recorded re-procedure during the follow-up period to avoid duplicate entries.

Medications were pooled into pharmacological groups based on Anatomical Therapeutic Chemical (ACT) codes [22]: antithrombotic agents (ATC code B01), antihypertensive agents (ATC codes C03, C07, C08, C09) and lipid modifying agents (ATC code C10). In Finland, SII reimburses medication purchases up to 3 months at a time (maximally 100 days covered). Medication adherence was defined as days covered on a yearly basis. The follow-up was divided into periods of 365 days beginning from patient discharge and until the end of the follow-up or patient death, whichever occurred first. A period of 365 days was considered adherent if the patient had purchased medication covering ≥ 292 days (80%) within that period and the interval between first and last purchase was ≥180 days. This correlates to an adherence rate of 80%, which is generally considered a measure of good adherence [23,24].

The principal investigator had unrestricted access to the data, maintained the database, prepared all drafts of the manuscript with all authors, and vouched for the integrity of the study.

## Ethics approval

All patients provided written informed consent. The Helsinki University Central Hospital Ethics

Committee approved the research protocol for the study (registry numbers 426/E5/05, 205/E0/2007, HUS/152/2016, and HUS/1203/2016). This study complies with the 1964 Declaration of Helsinki and subsequent revisions.

## Clinical endpoints

The primary objective was to determine the rate and location of new ischemic events in relation to target lesion stenting, and to differentiate factors related to the course of disease progression.

Secondary outcomes were major adverse cardiovascular events (MACE) defined as cardiovascular death, stroke, or repeat PCI.

## Statistical analysis

Data were reported as medians with interquartile ranges (IQRs) for continuous variables and as counts with percentages for categorical variables. Pairwise comparisons were conducted using the Mann–Whitney U-test for continuous variables in relation to outcomes, and the Chi-squared test for categorical variables in relation to outcomes. Logistic regression models were fitted to assess associations with different outcomes. Statistical significance was defined as a *p*-value <0.05. All analyses were performed using R software, version 4.5.0.

## Results

A total of 1970 patients were included in the study. Among the study population, 1335 patients presented with acute coronary syndrome (ACS) and 635 patients with chronic coronary syndrome (CCS). The majority of the patients were males. There were more patients diagnosed with diabetes and dyslipidaemia in the CCS group. Current smoking was more common among

ACS patients. The majority (71.0%) of implanted stents were BMS. Most DES (21.3%) were first-generation devices. Second-generation DES were used in 5.2% of cases, and in 2.4% of cases the stent type was unknown. The median length of stents was 20 (IQR 16–30) mm in ACS group and 20 (IQR 12–29) mm in CCS group (*p* = 0.161 between groups). Baseline characteristics are summarized in Table 1(A,B).

**Table 1A.** Patient characteristics during the initial percutaneous coronary intervention.

Variable	All *n* = 1970	ACS *n* = 1335	CCS *n* = 635	*P*-value
Age	64.8 (56.2–73.8)	64.0 (55.6–73.8)	65.8 (57.8–73.9)	0.021
Sex (women)	624 (31.7%)	422 (31.6%)	202 (31.8%)	0.970
**Past medical history**				
Diabetes	366 (18.6%)	201 (15.1%)	165 (26.0%)	<0.001
Dyslipidaemia	1393 (70.7%)	850 (63.8%)	543 (85.5%)	<0.001
Hypertension	1282 (65.1%)	836 (62.6%)	446 (70.2%)	0.001
Peripheral vascular disease	138 (7.0%)	88 (6.7%)	50 (7.9%)	0.373
Atrial fibrillation	102 (5.2%)	89 (6.7%)	13 (2.0%)	<0.001
Previous PCI	256 (13.0%)	112 (8.4%)	144 (22.7%)	<0.001
Smoking: Current	607 (30.8%)	471 (35.6%)	136 (21.4%)	<0.001
Smoking: Previous	641 (32.5%)	389 (29.4%)	252 (39.7%)	<0.001
**Clinical presentation**				
none	627 (31.8%)	2 (0.1%)	625 (98.4%)	<0.001
STEMI	577 (29.3%)	577 (43.2%)		
NSTEMI	624 (31.7%)	619 (46.4%)	5 (0.8%)	
UAP	142 (7.2%)	137 (10.3%)	5 (0.8%)	
**Laboratory findings**				
Triglycerides mmol/L	1.20 (0.91–1.67)	1.21 (0.93–1.68)	1.16 (0.87–1.67)	0.056
Lp(a) mg/L	9.9 (4.9–23.1)	10.3 (5.2–22.9)	9.4 (4.6–25.2)	0.243
HDL mmol/L	1.18 (1.01–1.40)	1.16 (1.01–1.37)	1.21 (1.02–1.47)	0.014
LDL mmol/L	2.82 (2.20–3.49)	3.03 (2.43–3.67)	2.46 (1.99–3.03)	<0.001
Cystatin C mg/L	1.21 (1.03–1.47)	1.18 (1.00–1.45)	1.25 (1.07–1.51)	<0.001
NT-proBNP ng/L	406 (152–1258)	644 (226–1716)	217 (109–528)	<0.001
**Stent type**				
BMS	1267 (64.3%)	918 (82.7%)	349 (73.6%)	
DES	249 (12.6%)	152 (13.7%)	97 (20.5%)	<0.001
BMS and DES both	68 (3.5%)	40 (3.6%)	28 (5.9%)	
Average stent length in mm	18 (12–24)	18 (15–24)	20 (12–28)	0.545
**Adherence to** **medication**				
**Statin**				
during 1st year	1518 (77.1%)	1025 (76.8%)	493 (77.6%)	0.714
overall adherence	1146 (58.2%)	747 (56.0%)	399 (62.8%)	0.004
**Antihypertensive**				
during 1st year	1278 (64.9%)	884 (66.2%)	394 (62.0%)	0.078
overall adherence	1210 (61.4%)	824 (61.7%)	386 (60.8%)	0.727
**Endpoints**				
Death	635 (32.2%)	425 (31.8%)	210 (33.1%)	0.619
CV-Death	253 (12.8%)	165 (12.4%)	88 (13.9%)	0.391
CV-event	530 (26.9%)	364 (27.3%)	166 (26.1%)	0.637
Stroke	216 (11.0%)	144 (10.8%)	72 (11.3%)	0.772
Myocardial infarction	364 (18.5%)	252 (18.9%)	112 (17.6%)	0.549
**Revascularizations**				
TL-Restenosis	137 (39.4%)	85 (41.7%)	52 (36.1%)	0.351
NTL-stenosis	212 (60.7%)	119 (58.3%)	92 (63.9%)	0.312
CABG	14 (0.7%)	13 (1.0%)	1 (0.2%)	0.084

Following the index procedure, 639 (32.4%) patients had at least one cardiovascular event, including 253 (12.8%) cardiovascular deaths. Of the patients 348 (17.7%) required repeat ischemia-driven revascularization at least once during follow-up. PCI was more frequent in CCS than in ACS patients (144 (22.7%) vs. 204 (15.3%), *p* = 0.001). On the contrary, CABG was borderline more frequent in ACS than in CCS (13 (1.0%) vs. 1 (0.2%), *p* = 0.084).

**Figure 1.**
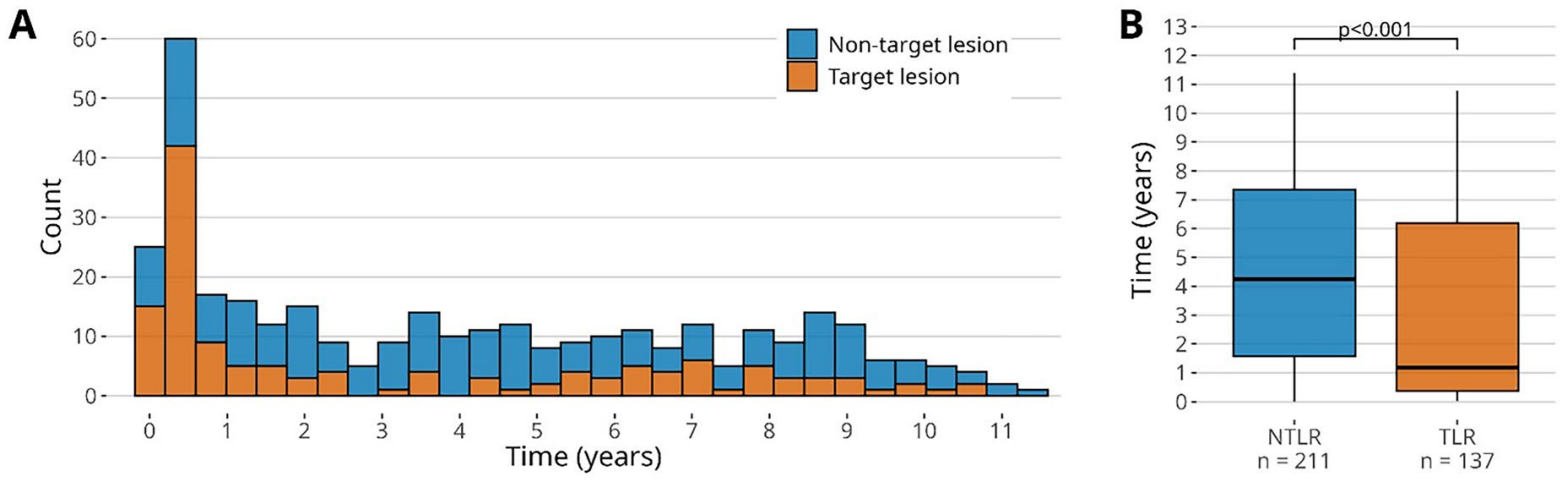
(A) Incidence non-target lesion and target lesion revascularizations during follow-up. (B) Box plot of time interval to revascularization. NTLR = new target lesion revascularization, TLR = target-lesion revascularization. Compared using Mann-Whitney U test.

Repeat revascularization occurred more frequently at new target lesions (NTLR) in 212 cases (60.7%) than at target lesions (TLR) in 137 cases (39.3%). Time to TLR was significantly earlier than time to NTLR (1.2, IQR 0.4–6.2 years vs 4.3, IQR 1.6–7.3 years, *p* < 0.001) (Figure 1). Risk factors associated with repeat revascularization included advanced age, higher NT-proBNP levels and multiple stents (Table 2(A)).

**Table 1B.** Characteristics of the patients with subsequent percutaneous intervention.

Variable	NTL and TL *n* = 349	NTL *n* = 212	TL *n* = 137	*P*-value
Age	64.0 (55.4–71.9)	63.0 (55.1–71.6)	65.1 (56.1–72.9)	0.396
Sex (women)	103 (29.5%)	59 (27.8%)	44 (32.1%)	0.461
**Past medical history**				
Diabetes	81 (23.2%)	42 (19.8%)	39 (28.5%)	0.082
Dyslipidaemia	253 (72.5%)	158 (74.9%)	95 (69.3%)	0.313
Hypertension	238 (68.2%)	148 (69.8%)	90 (65.7%)	0.491
Peripheral vascular disease	31 (8.9%)	14 (6.6%)	17 (12.4%)	0.095
Atrial fibrillation	13 (3.7%)	5 (2.4%)	8 (5.8%)	0.165
Smoking: Current	96 (27.5%)	61 (28.9%)	35 (25.5%)	0.369
Smoking: Previous	129 (37.0%)	72 (34.1%)	57 (41.6%)	
**Clinical presentation**				
none	138 (39.5%)	90 (42.5%)	48 (35.0%)	0.266
STEMI	76 (21.8%)	47 (22.2%)	29 (21.2%)	
NSTEMI	107 (30.7%)	62 (29.2%)	45 (32.8%)	
UAP	28 (8.0%)	13 (6.1%)	15 (10.9%)	
**Laboratory findings**				
Triglycerides mmol/L	1.23 (0.93–1.76)	1.25 (0.96–1.85)	1.17 (0.88–1.68)	0.075
Lp(a) mg/L	11.5 (5.6–32.5)	11.9 (6.0–35.6)	10.1 (5.3–24.6)	0.291
HDL mmol/L	1.17 (1.00–1.40)	1.15 (0.98–1.39)	1.22 (1.03–1.41)	0.094
LDL mmol/L	2.74 (2.15–3.39)	2.74 (2.20–3.47)	2.77 (2.07–3.18)	0.197
Cystatin C mg/L	1.23 (1.04–1.47)	1.24 (1.08–1.50)	1.17 (1.00–1.38)	0.023
NT-proBNP ng/L	296 (124–861)	292 (134–853)	330 (118–885)	0.820
**Stent type**				
BMS	207 (59.3%)	129 (72.9%)	78 (72.2%)	0.031
DES	55 (15.8%)	39 (22.0%)	16 (14.8%)	
BMS and DES both	23 (6.6%)	9 (5.1%)	14 (13.0%)	
Average stent length in mm	20 (15–30)	18 (12–26)	20 (16–32)	0.026
**Adherence to medication**				
**Statin**				
during 1st year	274 (78.5%)	175 (82.5%)	99 (72.3%)	0.032
overall adherence	214 (61.3%)	133 (62.7%)	81 (59.1%)	0.573
**Antihypertensive**				
during 1st year	236 (67.6%)	149 (70.3%)	87 (63.5%)	0.228
overall adherence	234 (67.0%)	149 (70.3%)	85 (62.0%)	0.138
**Endpoints**				
Death	98 (28.1%)	57 (26.9%)	41 (29.9%)	0.620
CV-Death	52 (14.9%)	33 (15.6%)	19 (13.9%)	0.779
CV-event	181 (51.9%)	115 (54.2%)	66 (48.2%)	0.318
Stroke	31 (8.9%)	21 (9.9%)	10 (7.3%)	0.520
Myocardial infarction	174 (49.9%)	112 (52.8%)	62 (45.3%)	0.203

ACS = acute coronary syndrome, BMS = bare metal stent, CABG = coronary artery bypass grafting, CCS = chronic coronary syndrome, CV = cardiovascular, DES = drug-eluting stent, HDL = High density lipoprotein cholesterol, LDL = low density lipoprotein cholesterol, Lp (a) = lipoprotein (a), NSTEMI = non-ST-elevation myocardial infarction, NTL = non-target lesion, NT- proBNP = N-terminal pro B-type natriuretic protein, PCI = percutaneous coronary intervention, STEMI = ST-elevation myocardial infarction, TL = target lesion, UAP = unstable angina pectoris. Values are medians (IQR) and counts (percentage in group). Compared using Mann-Whitney U tests and Chi-squared tests.

**Table 2.** Results from a multivariable logistic regression model for clinical endpoints.

	CV death		CV event	
Variable	OR (95%CI)	P-value	OR (95%CI)	P-value
**(A) CV death and CV Event results from adjusted models**				
Age (per 10 yr)	1.62 (1.32–2.01)	<0.001	1.14 (0.99–1.31)	0.073
Sex: female	0.97 (0.63–1.47)	0.880	1.02 (0.75–1.38)	0.894
Atrial fibrillation	1.20 (0.60–2.31)	0.603	1.31 (0.73–2.30)	0.361
Diabetes	2.18 (1.43–3.31)	<0.001	1.54 (1.11–2.13)	0.009
BMI	1.07 (1.03–1.11)	<0.001	1.04 (1.01–1.07)	0.009
Smoking: current	1.10 (0.63–1.91)	0.745	0.87 (0.61–1.24)	0.444
Smoking: ex-smoker	1.42 (0.91–2.23)	0.123	0.89 (0.65–1.23)	0.495
NTproBNP (logaritmized)	1.63 (1.41–1.90)	<0.001	1.14 (1.03–1.26)	0.010
CystatinC	1.21 (0.86–1.68)	0.275	1.15 (0.88–1.49)	0.307
Stent length (per 10 mm)	1.15 (1.00–1.31)	0.043	1.07 (0.96–1.18)	0.209
Statin adherence ≥80%	0.31 (0.17–0.58)	<0.001	0.33 (0.21–0.51)	<0.001
Antihypertensive med. Adherence ≥80%	1.11 (0.41–3.48)	0.852	0.44 (0.26–0.77)	0.004

**Table:** 

**(B) Restenosis data for target-lesion vs. non-target-lesion**				
Age (per 10 yr)	1.06 (0.87–1.29)	0.564	1.03 (0.75–1.42)	0.865
Sex: female	1.23 (0.77–1.96)	0.392	1.65 (0.86–3.18)	0.132
Atrial fibrillation	2.57 (0.84–8.65)	0.105		
Diabetes	1.61 (0.97–2.66)	0.063	2.07 (1.04–4.15)	0.038
BMI	0.98 (0.93–1.02)	0.313	0.96 (0.91–1.03)	0.256
Smoking: current	0.84 (0.52–1.37)	0.493	1.12 (0.54–2.32)	0.752
Smoking: ex-smoker	1.37 (0.88–2.13)	0.168		
NTproBNP (logaritmized)	1.02 (0.87–1.20)	0.808	0.90 (0.72–1.12)	0.346
CystatinC	0.57 (0.31–0.97)	0.046	0.59 (0.30–1.10)	0.111
Stent length (per 10 mm)	1.31 (1.09–1.58)	0.004	1.26 (1.02–1.55)	0.031
Statin adherence ≥80%	0.30 (0.19–0.47)	<0.001	0.45 (0.21–0.95)	0.036
Antihypertensive med. Adherence ≥80%	0.29 (0.18–0.48)	<0.001	0.67 (0.30–1.52)	0.338

BMI = body mass index, CV = cardiovascular, NT- proBNP = N-terminal pro B-type natriuretic protein, NTLR = non-target lesion revascularization, TLR = target-lesion revascularization, Revascularization model was simplified by dropping atrial fibrillation and previous smoking to avoid overfitting. *Calculation for the whole data, **Calculated for restenosis-data only.

Adherence to statin medication declined with time, as expected, but remained at a relatively high level even after 9 years **(**Supplementary Figures 1 and 2). During the first year after PCI 76.8% of patients in ACS group and 77.5% in CCS group had good adherence to statin medication (≥80% days covered) (*p* = 0.800 between groups). During the whole follow-up period 55.8% of patients in the ACS group and 63.3% in the CCS group had good adherence to statin medication (*p* = 0.002). The most prescribed statin was simvastatin (54.7%), followed by atorvastatin (27.7%) and rosuvastatin (7.5%). Most patients (74.4%) were receiving moderate-intensity, while 11.6% remained high-intensity, and the rest were on low-intensity statin therapy. During follow-up, statin therapy could have been modified by switching to other statins or adjusting the dose, either to intensify treatment or to reduce side effects (Supplementary Figures 3 and 4). Antihypertensive medication was used less conscientiously during the first year after PCI with 66.3% (ACS) and 61.8% (CCS) of patients adhering ≥80% to prescribed dosing. Adherence to antihypertensive medication persisted over the years. Among ACS patients 61.7% and 60.7% among CCS patients had good adherence to blood pressure lowering medication throughout the whole 10-year period. Adherence to anticoagulants was 74.7% (ACS) and 73.9% (CCS) during the first year of follow-up. We did not calculate antithrombotic adherence, since the period it was used varied due to multiple reasons, such as the stent type, usage of anticoagulation, and whether patient had ACS or CCS [25]. These findings are in accordance with previous studies investigating long-term medication adherence among Swedish patients after CABG [26].

Multivariate logistic regression models identified several risk factors associated with TLR compared to NTLR, including statin adherence, cumulative stent length, and diabetes (Table 2(B)).

High adherence to statin therapy (≥ 80% of time) significantly reduced the likelihood of TLR versus NTLR. Conversely, increased stent length and the presence of diabetes were associated with a greater risk of TLR compared to NTLR (Table 2(B)).

## Discussion

Main findings of this study are: (1) good adherence to statin therapy significantly reduces target lesion (TL)–related events more than non–target lesion (NTL)–related events; (2) both TL- and NTL-related ischemic events continue to occur for at least 10 years following initial coronary stenting; and (3) NTL-related events accounted for the majority of these repeat revascularizations.

Coronary atherosclerosis concerns the whole coronary tree, and despite secondary prevention measures, atherosclerosis will continue to advance in stented and non-stented locations. Longterm follow-ups have shown that non-target lesion events are more frequent than target lesion events [27,28]. The reason for that may be simply the fact that non-stented coronary volume is larger, and, therefore, the probability of new events is also higher. Accordingly, this study showed that new target lesion-related events accounted for most of these repeat revascularizations.

There has long been an expectation that coronary interventions could provide a definitive solution for coronary stenosis. While these procedures are lifesaving in the setting of acute coronary syndrome, significantly reducing mortality and the incidence of myocardial infarctions [29], their primary benefit in chronic coronary syndrome is symptom relief. The initial assumption that a stented coronary segment would be permanently ‘cured’ has not been fully realized, with stent restenosis remaining a major challenge.

Multiple interrelated factors are linked to stent restenosis: clinical factors include diabetes mellitus, elevated low-density lipoprotein levels, chronic kidney disease, heart failure, older age, and female sex; anatomic factors include vessel size and lesion characteristics; procedural factors include stent under expansion, and malposition; and stent-related factors include stent length, stent type and structure [11,28,30–32]. Likewise, in this study, we found that longer stents and diabetes were significant risk factors for stent restenosis.

Stents can also promote hypersensitivity and inflammatory reactions, which were more prominent in first-generation DES than BMS or second-generation DES^3^. These reactions explain the increased risk of late and very late stent thrombosis with first-generation DES [1,5]. With BMS, late lumen loss tends to peak 6–8 months after implantation and then decreases over time. The same trend could be seen in this study. Conversely, with DES, there is a slower but ongoing neointimal accumulation. Despite the substantial improvements in DES technologies, in-stent restenosis and the need for TLR still occur at 1%-2% per year, even with contemporary DES platforms^3^. This has been shown to continue for at least 5 years [7,10,27,30,33,34]. However, the literature extending beyond that is sparse [27,31,33]. The current study represents one of the longest follow-up periods published in TLR vs NTLR literature, extending the finding that TL restenosis continues for at least 10 years after initial coronary stenting. In our population, it is evident that atherosclerosis continues to progress also in native NTL sites despite the use of statins.

Adherence to evidence-based pharmacological therapies plays a crucial role in mitigating adverse events in both stable coronary artery disease and post-acute ischemic episodes [30,32,34–38]. We had extensive data on medication use throughout the follow-up period, and the extended 10-year follow-up allowed a comprehensive examination of the progression of coronary artery disease in both natural and stented segments in a real-world setting. Statins protected from all end points including target-lesion revascularizations, especially when used ≥80% of the time. This underscores the importance of lifetime adherence to lipid-lowering therapy, especially in diabetic patients with long or multiple stents. High-intensity LDL-lowering therapy may even promote plaque regression and stabilization, as observed in several coronary imaging studies [12]. However, its effect on long-term cardiovascular outcomes requires additional research. One example of that is proprotein convertase subtilisin/kexin type 9 inhibitor or its gene silencing, which may stabilize LDL cholesterol levels better than statins and that way prevent atherosclerosis progression [39].

Statin therapy after PCI can reduce the risk of repeat revascularization [40]. Less is known about the statin effect on TLR vs NTLR. One study shows that statins reduce TLR in sirolimus stents, but not in BMS [41]. Small, randomized studies have previously shown that statin does not reduce TLR vs target vessel revascularizations, however, no such studies on TLR vs NTLR have been published [42–44]. It should be noted that the target vessel means the same coronary artery where the initial stent is located, but the non-target lesion is a broader concept, including all locations and coronaries, but not the stent spot, and 5 mm proximal and 5 mm distal to the stent. In the present study, NTLR was more frequent than TLR, and good adherence to statin therapy reduced TLR more than NTLR.

Statins are known to reduce the risk of plaque rupture in native coronaries [45,46]. The direct evidence that statins will also reduce plaque rupture in in-stent neoatherosclerosis is lacking.

Neoatherosclerosis is frequently observed in patients with very late stent thrombosis, i.e. more than one year after stenting. This phenomenon is significantly associated with the implantation of drug-eluting stents. Furthermore, in-stent plaque rupture often occurs in patients with neoatherosclerosis and previous myocardial infarction [47]. Aligning with this finding, in the present study TLR was more frequent in ACS patients than in CCS patients. It is plausible that in-stent plaque rupture can be prevented with statin in a similar manner to native coronaries. The observed finding that statins reduce TLR may be attributed to statins’ pleiotropic effects, reducing neo-atherosclerosis through various mechanisms. Statin therapy reduces coronary inflammation [45]. However, there is also evidence that statins reduce stent-induced inflammation. In a porcine model, statin-coated stents were used, and they decreased neointimal hyperplasia [48].

## Limitations

The study cohort represents a large prospective material from a single center. However, the number of repeated revascularizations is moderate. Larger material in multiple centers is needed to confirm our findings. Since the study enrollment period, procedural techniques and pharmacologic therapies have advanced. In this cohort, bare metal stents were predominantly used, and clopidogrel was the primary antithrombotic agent following stenting, unlike current practice, using more contemporary DES technologies, and frequently ticagrelor for post-ACS management.

In cases of death or conservatively managed ischemic events, the exact location of potential coronary stenoses was not determined, which may have influenced the proportions and endpoints related to target lesions (TL) and new target lesions (NTL).

The study population represents prospectively collected registry data. Therefore, medication adherence has been obtained from purchase history, but the actual use of the medication cannot be determined. Additionally, the use of acetylsalicylic acid is not known since it is mostly bought over the counter. Because follow-up relies on registry data, we lack detailed information on several clinically relevant aspects. Moreover, no patient-level data are available beyond the index hospitalization; for example, LDL levels during follow-up are unknown.

## Conclusion

Ischemic events related to both TL and NTL persist for at least 10 years following initial coronary stenting. Notably, the majority of repeat revascularizations during this period are driven by NTL-related events. Achieving over 80% adherence to statin therapy provides significantly greater protection against TLR compared to NLR.

Previous long-term lipid-lowering randomized controlled trials in stented patients with imaging data could help validate our findings. Stent trials with extended follow-up and varied lipid-lowering regimens may also offer insights into in-stent neoatherosclerosis. Ultimately, the most robust approach to confirm the reproducibility of our results would be a dedicated randomized controlled trial incorporating intravascular imaging and long-term follow-up.

## Supplementary Material

Supplementary_material.docx

## Data Availability

The data of this study will be made available on reasonable request from the corresponding author.
