# Supplementary material for: Lipid-lowering medication reduces more target lesion than non-target lesion revascularizations: a cohort study
Source: Ann Med. 2026 Feb 12;58(1):2625586. doi: 10.1080/07853890.2026.2625586 (PMC12912242; doi:10.1080/07853890.2026.2625586)

**Supplementary material: Mantyniemi Elina et al.**

**Supplementary Figure 1.** Statin adherence during the whole study in all patients (n=1970).

**Supplementary Figure 2.** Statin adherence during the whole study in **A)** non-target lesion revascularization (n=212) and **B)** target lesion revascularization patients (n=137).

**Supplementary Figure 3A.** Statin use at baseline on all patients (n=1970), according to different molecules and lipid-lowering intensity.

**Baseline**

% of purchases

**Supplementary Figure 3B.** Statin use during follow-up on all patients (n= 1970), according to different molecules and lipid-lowering intensity.

**Follow up**

% of purchases

**Supplementary Figure 4.** Statin use at baseline and during follow-up, according to different molecules and lipid-lowering intensity.

1. On non-target-lesion revascularization (NTLR) patients (n=212)
2. On target-lesion revascularization (TLR) patients (n=137)
3. On all NTLR +TLR patients (n=349)


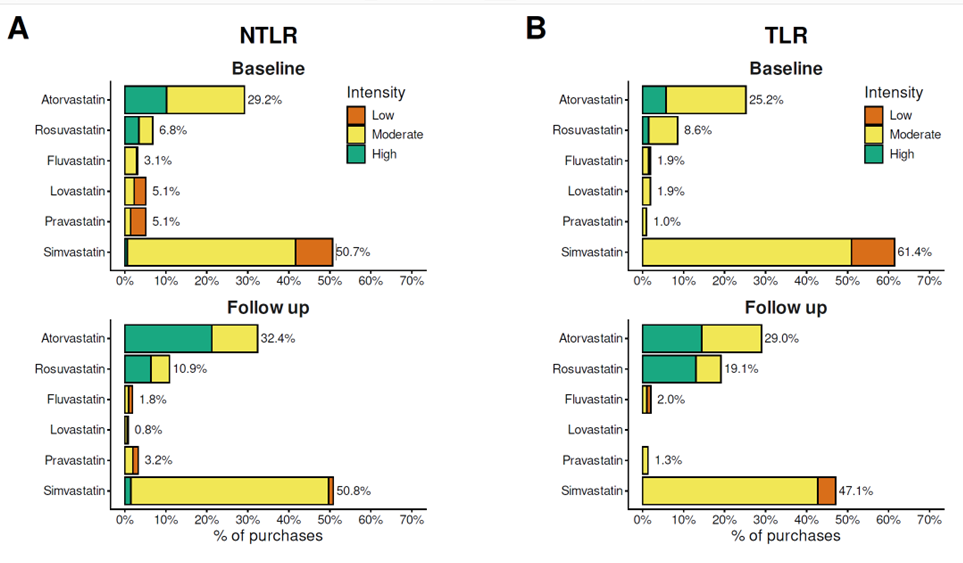


**C**


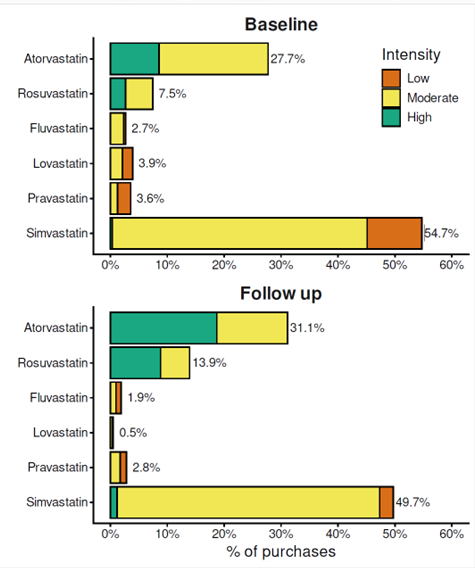

Supplement: Supplementary_material.docx [file IANN_A_2625586_SM6110.docx]
